# Minimal Elements Required for the Formation of Respiratory Syncytial Virus Cytoplasmic Inclusion Bodies *In Vivo* and *In Vitro*

**DOI:** 10.1128/mBio.01202-20

**Published:** 2020-09-22

**Authors:** Marie Galloux, Jennifer Risso-Ballester, Charles-Adrien Richard, Jenna Fix, Marie-Anne Rameix-Welti, Jean-François Eléouët

**Affiliations:** a Université Paris-Saclay, INRAE, UVSQ, VIM, Jouy-en-Josas, France; b Université Paris-Saclay, INSERM, Université de Versailles St. Quentin, UMR 1173 (2I), Versailles, France; c AP-HP Université Paris Saclay, Hôpital Ambroise Paré, Laboratoire de Microbiologie, Boulogne-Billancourt, France; Columbia University Medical College

**Keywords:** RSV, inclusion bodies, liquid-liquid phase, nucleoprotein, phosphoprotein, protein-protein interactions

## Abstract

Infection of host cells by the respiratory syncytial virus (RSV) is characterized by the formation of spherical cytoplasmic inclusion bodies (IBs). These structures, which concentrate all the proteins of the polymerase complex as well as some cellular proteins, were initially considered aggresomes formed by viral dead-end products. However, recent studies revealed that IBs are viral factories where viral RNA synthesis, i.e., replication and transcription, occurs. The analysis of IBs by electron microscopy revealed that they are membrane-less structures, and accumulated data on their structure, organization, and kinetics of formation revealed that IBs share the characteristics of cellular organelles, such as P-bodies or stress granules, suggesting that their morphogenesis depends on a liquid-liquid phase separation mechanism. It was previously shown that expression of the RSV nucleoprotein N and phosphoprotein P of the polymerase complex is sufficient to induce the formation of pseudo-IBs. Here, using a series of truncated P proteins, we identified the domains of P required for IB formation and show that the oligomeric state of N, provided it can interact with RNA, is critical for their morphogenesis. We also show that pseudo-IBs can form *in vitro* when recombinant N and P proteins are mixed. Finally, using fluorescence recovery after photobleaching approaches, we reveal that *in cellula* and *in vitro* IBs are liquid organelles. Our results strongly support the liquid-liquid phase separation nature of IBs and pave the way for further characterization of their dynamics.

## Abstract (precis)

Respiratory syncytial virus (RSV) is the leading cause of lower respiratory tract illness in infants, elderly, and immunocompromised people. No vaccine or efficient antiviral treatment is available against this virus. The replication and transcription steps of the viral genome are appealing mechanisms to target for the development of new antiviral strategies. These activities take place within cytoplasmic inclusion bodies (IBs) that assemble during infection. Although expression of both the viral nucleoprotein (N) and phosphoprotein (P) allows induction of the formation of these IBs, the mechanism sustaining their assembly remains poorly characterized. Here, we identified key elements of N and P required for the scaffolding of IBs and managed for the first time to reconstitute RSV pseudo-IBs *in vitro* by coincubating recombinant N and P proteins. Our results provide strong evidence that the biogenesis of RSV IBs occurs through liquid-liquid phase transition mediated by N-P interactions.

## INTRODUCTION

The human respiratory syncytial virus (RSV) is the leading cause of severe respiratory tract infections in newborn children worldwide (1). It infects close to 100% of infants within the first 2 years, and it is the main cause of bronchiolitis. A recent study on the etiology of pneumonia in children hospitalized in seven countries revealed that RSV is responsible for 31% of the cases of acute respiratory infections (http://perchresults.org/). Furthermore, RSV is also recognized as a significant cause of severe respiratory infections in the elderly and in immunocompromised patients (1). Although it has a huge impact on human health, there is still no vaccine against human RSV. The only treatment available is the preventive injection to at-risk young children of a monoclonal antibody (Palivizumab) that presents a low cost-benefit ratio (2).

RSV belongs to the *Mononegavirales* (MNV) order and constitutes the prototype virus of the *Pneumovirus* genus of the *Pneumoviridae* family (3). The viral genome is a nonsegmented negative-strand RNA that is enwrapped by the nucleoprotein (N), forming a helical nucleocapsid (4). This ribonucleoprotein complex (RNP) serves as the template for viral transcription and replication by the viral polymerase (5). RSV replicates in the cytoplasm of host cells, and infection induces the formation of spherical cytoplasmic granules called inclusion bodies (IBs). Similar structures called Negri bodies were initially observed upon rabies virus (RABV) infection and constituted a signature for the diagnosis of the infection (6, 7). Since then, it appeared that IBs are a hallmark of infection for many other MNVs such as vesicular stomatitis virus (VSV) (8), measles virus (MeV) (9), metapneumovirus (MPV) (10), Ebola virus (11), Marburg virus (12), Nipah virus (13), and parainfluenza virus (PIV) (14, 15). It was shown that these structures are viral factories where all the viral proteins of the polymerase complex concentrate to perform the replication and transcription of the viral genome (16). For RSV, it was also shown that other viral proteins such as the nonstructural protein NS2 and the matrix protein M can be recruited to IBs during the virus life cycle (17, 18). Similarly, cellular proteins that could be involved in mRNA translation, in the activity of the polymerase complex, the dynamics of IBs, or in the control of host immune response were shown to concentrate within IBs. More specifically for RSV, the poly(A)-binding protein (PABP) and the translation initiation factor eIF4G (19), cellular proteins involved in posttranslational modifications such as the phosphatase PP1 (protein phosphatase 1) (20), the chaperones HSP90 and HSP70 (21, 22), actin and actin-associated proteins (23, 24), and the proteins involved in antiviral responses MDA5 (melanoma differentiation-associated gene 5) and MAVS (mitochondrial antiviral signaling) (25) were shown to colocalize within IBs.

Although IBs play a key role during the life cycle of viruses, and could represent targets of choice for the development of new antiviral strategies, their morphogenesis, dynamics, and molecular organization still remain poorly characterized. Electron microscopy studies of rabies virus-infected cells revealed that these structures are spherical membrane-less inclusions (26). Using time-lapse fluorescence microscopy, it was shown that IBs formed upon MeV, VSV, RABV, and RSV infection are highly dynamic and that they can fuse and deform, suggesting that they display the characteristics of membrane-less inclusions that could be generated from liquid-liquid phase separation (9, 19, 27). Finally, it was recently shown that RSV IBs present dynamic subcompartments called IBAGs (for IB-associated granules) where viral mRNA and the viral transcription factor M2-1 accumulate (19), suggesting a high degree of organization within these structures.

Although all the proteins of the RSV polymerase complex concentrate within IBs, the expression of N and P alone is sufficient to induce the formation of these structures (28). It is thus expected that the interactions between these two viral proteins are at the origin of the scaffold required for the morphogenesis of IBs. Like all the viruses belonging to the MNV order, there are two types of N-P complexes during viral cycle, which involve specific domains of interaction: the N^Nuc^-P complex (N:RNA-P interaction) required for the recruitment of L to RNPs; and the N^0^-P complex where P plays the role of chaperone to maintain the neosynthesized N monomeric and RNA-free, competent for the encapsidation of the viral antigenome and genome (29). The crystal structure of recombinant RSV N protein (391 residues) expressed in Escherichia coli and purified as rings of 10 N protomers and a 70-nucleotide-long RNA showed that N has two globular domains (N_NTD_ and N_CTD_) that form the RNA groove, and N- and C-arms involved in N oligomerization (30). The RSV P protein is composed of 241 amino acids, and it forms homotetramers of elongated shape. This protein has a modular organization with a central coiled-coil domain involved in oligomerization, flanked by two intrinsically disordered regions (IDRs) (positions 1 to 130 and 152 to 241) (31, 32). A nuclear magnetic resonance (NMR) study of P recently revealed that the N- and C-domains adopt transient secondary structures in solution (33) and that several of these transient regions correspond to domains of interaction of P with N^0^, M2-1, and L (20, 31, 34, 35). The interactions between N and P are also well described. For the interaction with N^Nuc^, the C-terminal extremity of P (residues 233 to 241) was shown to interact with a well-defined pocket localized at the surface of the N_NDT_ (36–38). More recent data revealed that within the N^0^-P complex, the 30 N-terminal residues of P bind at the surface of the N_CTD_ (34, 39).

In the present study, our aim was to gain information on the mechanisms of IB biogenesis. We investigated the relative role of N^0^-P versus N^Nuc^-P interactions in this process and characterized the minimal domains of P required for the formation of pseudo-IBs. Using recombinant N and P, we managed to reconstitute for the first time RSV pseudo-IBs *in vitro* and showed that IBs are liquid organelles. Our results provide strong evidence that the biogenesis of RSV IBs occurs through liquid-liquid phase transition mediated by N-P interactions.

## RESULTS

### The formation of IBs depends on the capacity of N to interact with RNA and/or to oligomerize.

We have previously shown that mutations of either P or N proteins targeting residues involved in the interaction between P and N^Nuc^ impaired IB formation (37). Similarly, mutations of N residues involved in N oligomerization induced a defect of IB formation (39).

**FIG 1:**
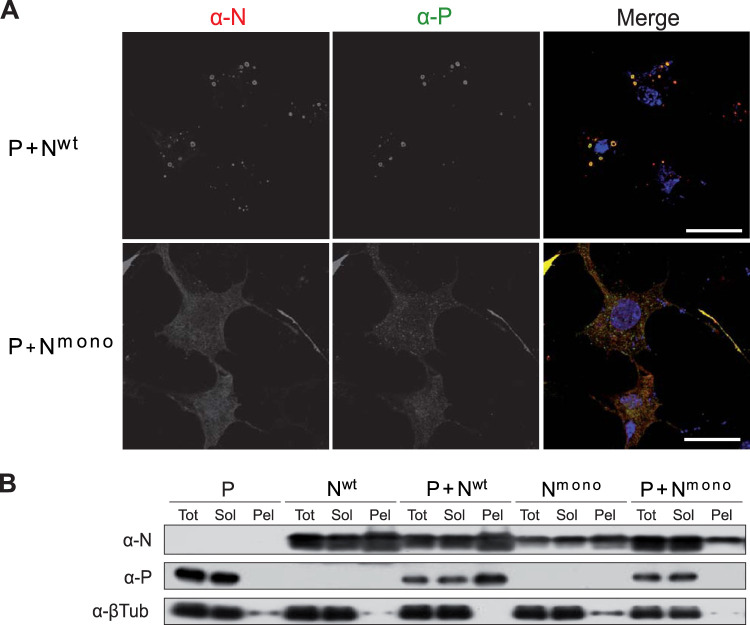
Study of P-N interactions in cells by immunofluorescence and soluble-insoluble fractionation. (A) Observation of cellular localization of P and N^wt^ or N^mono^ in cells. N and P proteins were coexpressed in BSRT7/5 cells. The cells were then fixed 24 h posttransfection and labeled with anti-P (α-P) (green) and anti-N (α-N) (red) antibodies, and the distribution of viral proteins was observed by fluorescence microscopy. Nuclei were stained with Hoechst 33342. Bars, 10 μm. (B) BSRT7/5 cells expressing P, N^wt^, or N^mono^ proteins or coexpressing P and N^wt^ or N^mono^ were lysed 24 h posttransfection, and the presence of P and N proteins in total (Tot), soluble (Sol), and insoluble (pellet [Pel]) fractions were analyzed by Western blotting.

To gain information on the respective roles of P-N^Nuc^ and P-N^0^ complexes for IB morphogenesis, we first compared the cellular localization of wild-type N (N^wt^) and of the N mutant K170A/R185A (N^mono^), unable to bind RNA and previously shown to mimic N^0^ in the presence of P (34). Cells were transfected with plasmids encoding P and N for 24 h, fixed, and stained with anti-N and anti-P antibodies before analyzing their localization by fluorescence microscopy. As shown in Fig. 1A, coexpression of N^wt^ and P allowed us to observe cytoplasmic inclusions similar to those seen during viral infection. In contrast, when coexpressed, both N^mono^ and P mainly presented a diffuse cytoplasmic distribution, although small cytoplasmic aggregates of proteins were observed (Fig. 1A).

We then studied the solubility of P and N^wt^ or N^mono^ when expressed either individually or coexpressed, in order to further characterize N-P interactions under these conditions. Twenty-four hours after transfection, cell lysates were clarified, and the presence of N and P proteins in the supernatant and pellet fractions was analyzed by Western blotting using specific antibodies (Fig. 1B). When expressed alone, P was recovered only in the soluble fraction. When expressed alone, N^wt^ was mainly recovered in the pellet fraction. This is most likely due to the fact that N^wt^ associates with RNA, forming high-molecular-weight complexes. Cotransfection of cells with equal quantities of P- and N^wt^-encoding plasmids did not result in a significant solubilization of the N protein, and the P protein was mainly recovered in the pellet fraction. Thus, in this context, P failed to stably maintain N as a soluble form and interacts with N^Nuc^. On the other hand, expression of N^mono^ alone was characterized by an overall decrease in the N content compared to the level of expression of N^wt^, and N^mono^ was recovered in similar proportions in both the soluble fraction and the pellet. When coexpressed with P, N^mono^ was predominantly recovered in the soluble fraction, together with P. The absence of P in the pellet fraction suggests that N^mono^ alone is prone to aggregate, either due to a propensity to oligomerize or to misfold when overexpressed. These results suggest that in cells P acts as a chaperone, stabilizing N^mono^, and that the N^0^-P complex is mainly soluble in eukaryotic cells.

Altogether, our data show that the interaction between P and N monomers is not sufficient to induce the assembly of IBs, which seems to depend on the presence of N^Nuc^, competent for RNA encapsidation and oligomerization.

### Identification of the domains of P required for the morphogenesis of IBs.

**FIG 2:**
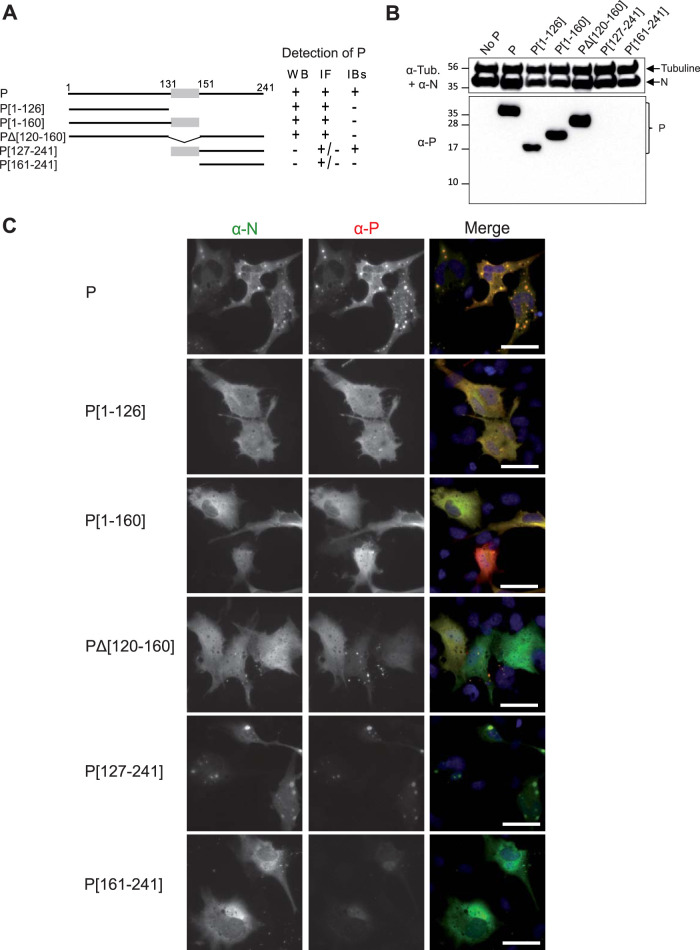
Identification of the domains of P required for the formation of IBs in cells. (A) Schematic illustration of truncated P proteins. The oligomerization domain of P is represented in gray, and numbers indicate amino acid positions. For each deletion mutant, the detection of P by Western blotting (WB), immunolabeling (immunofluorescence [IF]), and within IBs, by the rabbit polyclonal antibody, is summarized to the right of the schematic. (B) Western blot analysis of the expression of N and P fragments in BRST/7 cells cotransfected with pN and pP (or fragments). Detection of tubulin was used as a control. (C) Cellular localization of N^wt^ and P fragments in cells. N and P proteins were coexpressed in BSRT7/5 cells. The cells were then fixed 24 h posttransfection and labeled with anti-N (green) and anti-P (red) antibodies, and the distribution of viral proteins was observed by fluorescence microscopy. Nuclei were stained with Hoechst 33342. Bars, 20 μm.

We then investigated the role of P in the morphogenesis of IBs. More specifically, our aim was to determine the potential role of the different domains of P, i.e., N- and C-terminal IDRs and the oligomerization domain (OD), in this process. We thus generated deletions of each subdomain of P (Fig. 2A) and studied the impact of these deletions on IB formation. BSRT7/5 cells were cotransfected with the plasmid encoding N^wt^ together with full-length P or the different deleted P constructs. Expression of the proteins was then validated by Western blotting, and the cellular localization of N and P was studied by immunolabeling of fixed cells. In both cases, a rabbit polyclonal anti-P was used. As shown in Fig. 2B, P was detected with an apparent molecular weight of 35 kDa, instead of 27 kDa, as expected (28, 40). Similarly, the fragments P with residues 1 to 126 (P[1-126]), P[1-160], and P with residues 120 to 160 deleted (PΔ[120-160]), with sizes of 14 kDa, 18 kDa, and 23.5 kDa, respectively, were detected by Western blotting and migrated with higher apparent molecular weights than expected. In contrast, the fragments P[127-241] and P[161-241], with theoretical masses of 13 kDa and 9 kDa, respectively, were not detected. The absence of signal for these two fragments is mainly due to a lack of recognition by anti-P antibodies. Indeed, it was previously shown that most of the antibodies directed against P recognize the N-terminal part of P (28), the C-terminal domain being poorly immunogenic. However, although weak, a fluorescent signal was detected by immunolabeling with anti-P serum in cells expressing these two fragments (Fig. 2C, two bottom panels), suggesting that both fragments were expressed. Analysis of the cellular localization of N and P by fluorescence microscopy revealed that IBs similar to those formed in the presence of full-length P were observed only upon coexpression of N and P[127-241] (Fig. 2C). For all the other P fragments, N and P presented a diffuse cytoplasmic distribution.

**FIG 3:**
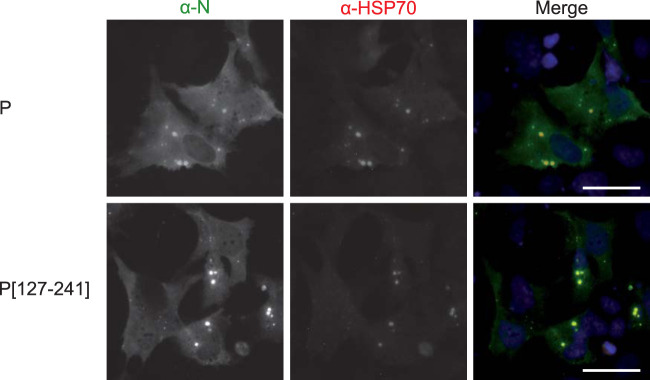
Analysis of the recruitment of HSP70 to IBs. Cells were cotransfected with N and P or P[127-241], fixed 24 h posttransfection, and labeled with anti-N (green) and anti-HSP70 (red) antibodies before observation by fluorescence microscopy. Nuclei were stained with Hoechst 33342. Bars, 20 μm.

We then investigated the capacity of the pseudo-IBs observed upon coexpression of N with wild-type P or P[127-241] to recruit cellular proteins. We then tried to detect the presence of HSP70, which was previously shown to interact with both RSV N and P (21, 41). As shown in Fig. 3, in cells cotransfected with pN and pP or pP[127-241] and immunolabeled with anti-N and anti-HSP70, endogenous HSP70 concentrates in IBs in both cases.

Thus, it appears that deletion of the N-terminal IDR of P does not impact IB morphogenesis and that both the oligomerization domain and the C-terminal IDR of P are required to induce the formation of IBs similar to those formed in the context of full-length P.

### Role of the C-terminal domain [160-227] of P in the formation of IBs.

**FIG 4:**
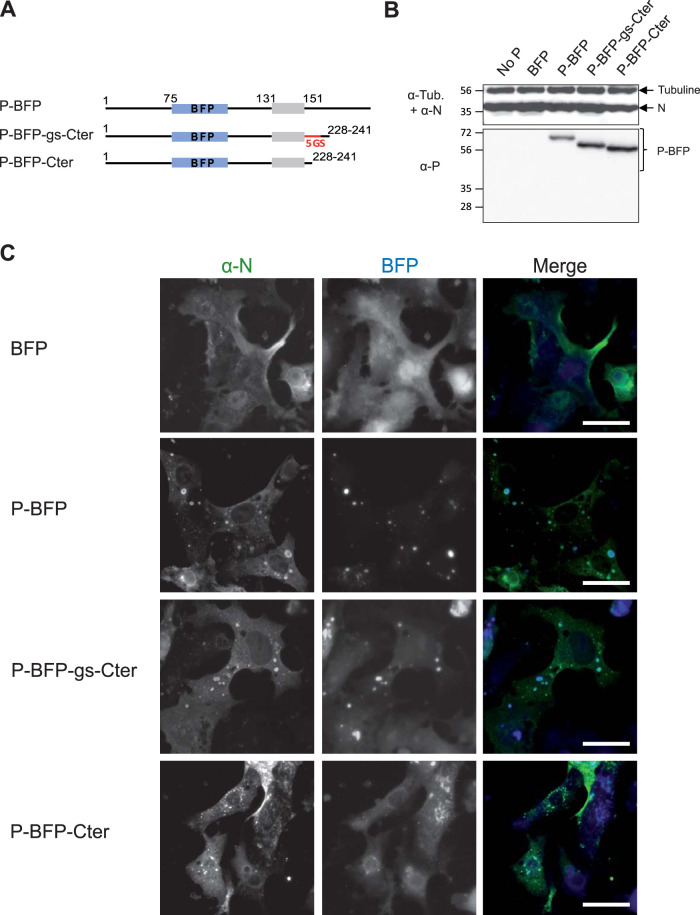
Study of the role of the intrinsically disordered domain [160-227] of P for IB formation. (A) Schematic illustration of the full-length and truncated P-BFP proteins. The oligomerization domain of P is represented in gray. The BFP is shown in blue, and the sequence of five Gly-Ser repetition is shown in red. Numbers indicate amino acid positions. (B) Western blot analysis of the expression of N and P-BFP proteins in BRST/7 cells cotransfected with pN- and pP-BFP-derived constructs. Detection of tubulin was used as a control. (C) Cellular localization of N and P-BFP proteins in cells. N and P-BFP proteins were coexpressed in BSRT7/5 cells. The cells were then fixed 24h posttransfection and labeled with anti-N (green) antibodies, and the distribution of viral proteins was observed by fluorescence microscopy. Bars, 20 μm.

We previously determined that the last nine C-terminal residues of P involved in the interaction with N^Nuc^ are required for IB formation (36, 37), and we found here that the OD is also required. These two domains are separated by a highly conserved region among pneumoviruses corresponding to RSV P residues 160 to 180. Although this region is dynamic, the structural study of isolated P by NMR revealed that it presents two regions displaying high α-helical propensity (αC1 [Leu173-Met187] and αC2 [Asn189-Lys205]) (33) and that the αC1 could be a secondary P binding site to N. One key issue was thus to determine whether this domain is involved in secondary interactions with N required for the scaffolding of IBs, or based on its flexibility, if it mainly plays the function of a linker needed to induce the assembly of IBs. To further study the role of this P domain on IB formation, our approach was to investigate whether residues 161 to 232 ([161-232]) could be deleted or replaced by a fully disordered spacer. Since the region [161-241] was poorly detected by polyclonal or monoclonal antibodies, we opted for a new strategy using the fluorescent P-BFP construct, the blue fluorescent protein (BFP) being inserted between residues 74 and 75 of P. We previously showed that insertion of BFP at this position does not impact the formation of IBs or the activity of the RSV polymerase complex (20). Using this P-BFP construct, the domain [160-227] was either substituted by a stretch of 5 Gly-Ser residues (P-BFP-gs-Cter) or deleted without introducing any linker between the OD and the last C-terminal residues of P (P-BFP-Cter) (Fig. 4A). Cells were cotransfected with the plasmids encoding N^wt^ and the P-BFP constructs, and the expression of the proteins was validated by Western blotting using an anti-P rabbit polyclonal serum (Fig. 4B). In parallel, transfected cells were fixed 24 h posttransfection and immunostained with anti-N antibody. As already shown, coexpression of P-BFP with N led to the formation of IBs similar to those observed with P, whereas no inclusions were observed when N was coexpressed with BFP alone (Fig. 4C). A defect of IB assembly was evidenced when P-BFP-Cter and N were coexpressed; N was more diffuse in the cytoplasm and also formed small cytoplasmic granules, while the P-BFP-Cter mostly presented a diffuse cytoplasmic distribution. In contrast, coexpression of N with the P-BFP-gs-Cter construct still allowed us to observe cytoplasmic inclusions similar to those seen with wild-type P. These results indicate that the fusion of the OD to the C-terminal residues of P, by deleting the region [160-227], impaired the architectural organization required for IB morphogenesis. However, substitution of this region by a short flexible linker can restore the capacity of P to induce the formation of IBs in the presence of N.

Altogether, our results show that the domain P[160-227] plays a critical role in the morphogenesis of IBs, mostly by creating a flexible linker between the OD and C terminus of P.

### *In vitro* reconstitution of pseudo-IBs.

**FIG 5:**
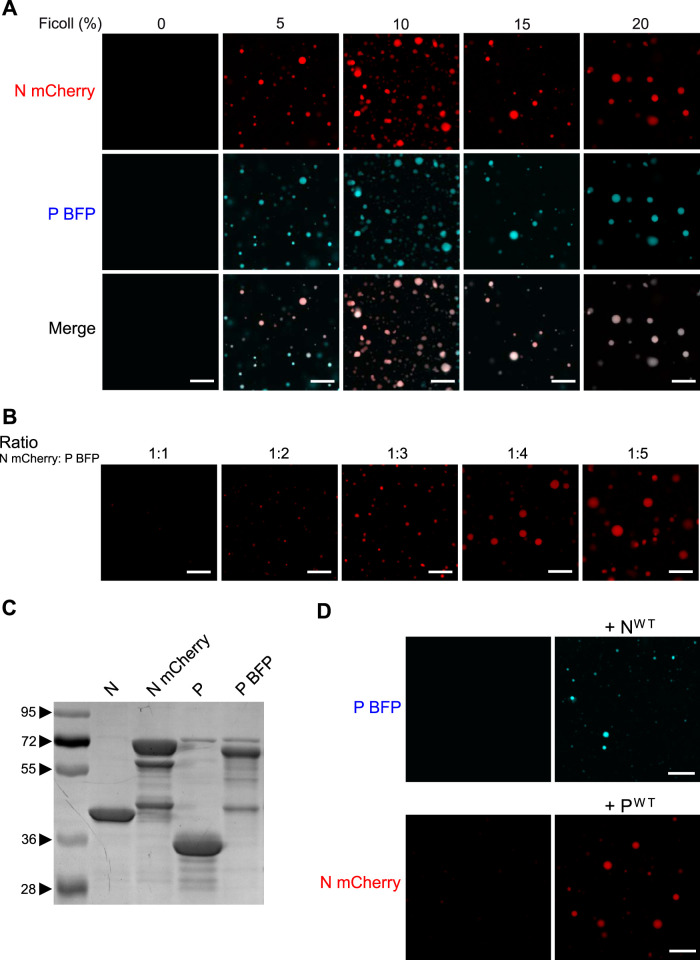
Reconstitution of pseudo-IBs *in vitro*. Recombinant N and P proteins were coincubated, and the analysis of phase separation was assessed using fluorescence microscopy. (A) Coincubation of mCherry-N (3 μM) and P-BFP (14 μM) in the presence of increasing concentratiosn of Ficoll. (B) Coincubation of mCherry-N (3 μM) and increasing concentration of P-BFP (from 3 μM to 14 μM) in the presence of 15% Ficoll. The ratio of mCherry N to P-BFP is indicated. (C) Analysis of purified N, mCherry-N, P, and P-BFP recombinant proteins by SDS-PAGE and Coomassie blue staining. (D) Coincubation of P-BPF and N^wt^ or mCherry-N and P^wt^ in the presence of 15% Ficoll. Bars, 10 μm.

As N^Nuc^ and P are sufficient to induce the formation of pseudo-IBs in cells, and based on data from the literature showing that cytoplasmic inclusions formed upon infection with other single-stranded negative-strand RNA viruses (e.g., rabies virus, VSV, and measles virus) exhibit the properties of membrane-less liquid organelles (9, 26, 42), we then investigated whether N^Nuc^ and P interactions could induce liquid-liquid phase separation *in vitro*. It was observed that when solutions of macromolecules undergo liquid-liquid phase separation, they condensate into a dense phase that often resembles liquid droplets (43). To study the phase separation *in vitro*, our approach was to generate recombinant fluorescent N and P proteins to observe the potential formation of droplets using fluorescence microscopy. We thus generated and purified recombinant P-BFP and mCherry-N proteins (Fig. 5C). As previously mentioned, the purified recombinant N corresponds to rings of 10 or 11 protomers interacting with RNA (30). To analyze droplet formation, recombinant mCherry-N and P-BFP were coincubated, using various P/N molecular ratios, on a glass slide in the presence of the molecular-crowding agent Ficoll (44), and fluorescence was observed by microscopy. In the absence of Ficoll, both mCherry-N and P-BFP presented a diffuse distribution (Fig. 5A, leftmost panels). We showed that addition of Ficoll (by increments of 5%, 10%, 15%, or 20% Ficoll) induced the formation of droplets of approximatively 1 to 5 μm in diameter, where both mCherry-N and P-BFP colocalized (Fig. 5A). These data were obtained using a P/N molecular ratio close to 4, as we determined that a P/N ratio of ≥4 allowed us to clearly observe IBs (Fig. 5B). To exclude the potential impact of fluorescent tags on droplet formation, we coincubated in parallel P-BFP with untagged N and mCherry-N with untagged P, in the presence of 15% Ficoll. As shown in Fig. 5D, both combinations of proteins allowed us to observe droplets. Our results thus reveal for the first time that RSV N and P are able to condensate into droplets, strongly suggesting that N complexed with RNA and P are the scaffold proteins driving RSV IB formation through liquid-liquid phase separation (LLPS).

**FIG 6:**
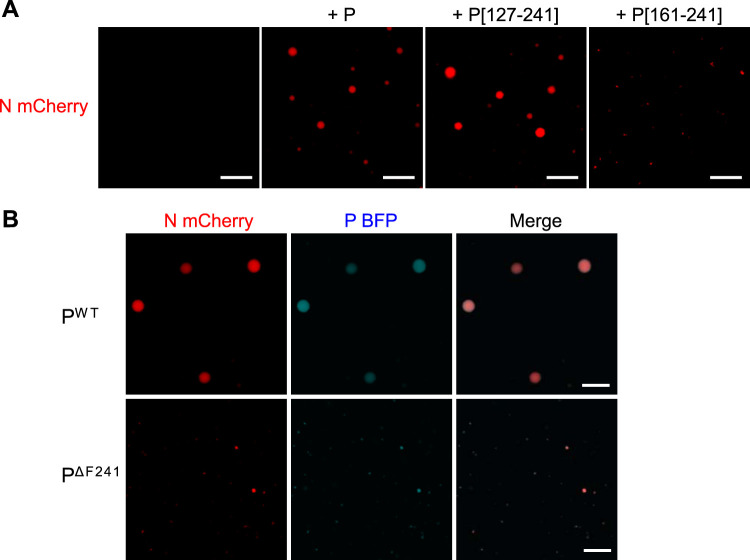
Validation of the minimal domains of P required for pseudo-IB formation *in vitro*. Recombinant N and P proteins were coincubated, and the analysis of phase separation was assessed using fluorescence microscopy. (A) Coincubation of mCherry-N (3 μM) with recombinant P or the fragments P[127-241] and P[161-241] (35 μM), 15% Ficoll; (B) coincubation of mCherry-N with recombinant P^wt^-BFP or P ^ΔF241^-BFP. Bars, 10 μm.

On the basis of our previous results in transfected cells showing that the fragment P[127-241] is sufficient to induce the formation of IBs, we then tested the potency of this P fragment to induce droplets in the presence of mCherry-N *in vitro* and used fragment P[161-241] as a control. As shown in Fig. 6A, mCherry-N formed similar droplets in the presence of P or P[127-241]. In contrast, mCherry-N mainly remained diffuse in the presence of P[161-241], with only few small condensates detectable. These results confirm that the N-terminal part of P is not required for the phase separation and that the oligomerization of P plays a critical role in IB morphogenesis. Finally, we tested the impact of the deletion of the C-terminal residue of P (F241) and generated a recombinant P-BFP^ΔF241^ protein. When coincubated with mCherry-N, both P-BFP^ΔF241^ and mCherry-N presented a diffuse fluorescence (Fig. 6B), confirming that the interaction between the C termini of P and N plays a critical role in the scaffolding required to induce droplet formation.

**FIG 7:**
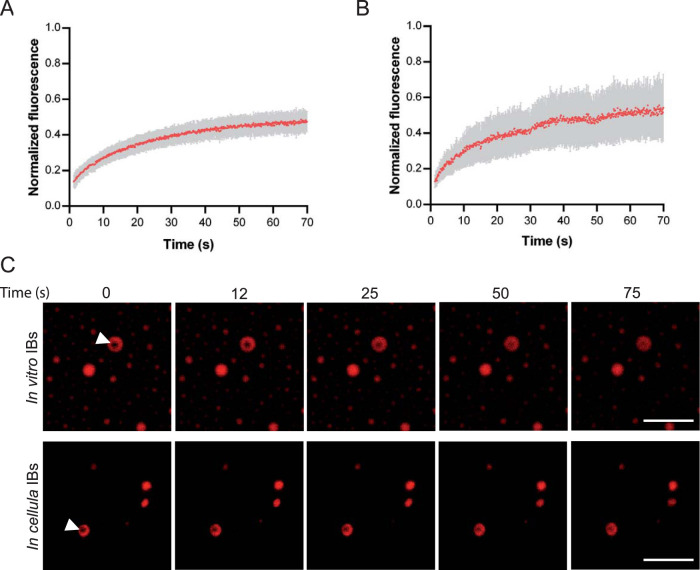
Study of IB viscosity. FRAP analysis of mCherry-N fluorescence in IBs. (A and B) Spontaneous redistribution of mCherry-N fluorescence after photobleaching on *in vitro* (A) and cellular (B) IBs was recorded, corrected (background and bleaching during postbleach imaging) and normalized to the average of the prebleach signal. Data are from 20 FRAP events recorded in two independent experiments. The mean of each experimental condition is shown with error bars representing standard deviations (SD). (C) Time-lapse images of FRAP on *in vitro* IBs (top panels), and on pseudo-IBs formed in cells transfected with pmCherry-N and pP for 24 h (bottom panels). Bars, 10 μm.

In order to confirm that RSV IBs are liquid organelles, we then performed fluorescence recovery after photobleaching (FRAP) experiments on *in vitro*-reconstituted IBs, using mCherry-N and P. We observed recovery of the mCherry fluorescence consistent with rapid diffusion of N in IBs (Fig. 7A and C). We then performed FRAP experiment on pseudo-IBs observed in cells upon expression of mCherry-N and P and showed that N diffusion was similar in pseudo-IBs compared to *in vitro* IBs (Fig. 7B and C). Of note, only 50% of recovery was observed. The fluorescence intensity being attenuated but homogenous in the whole IB, this could suggest the existence of an extremely mobile fraction destroyed during the bleach. The properties of N and P diffusion would need further investigation.

Altogether, our results strongly suggest that N and P assemble in biomolecular condensates exhibiting properties of liquid organelles that can be reconstituted *in vitro* by coincubation of recombinant N-RNA and P proteins.

## DISCUSSION

One of the hallmarks of infection by MNVs is the formation of membrane-less IBs in the cytoplasm of host cells. Since these structures have been shown to be viral factories, where replication and transcription take place, characterization of their nature, organization, and functioning constitutes a major issue to better understand the mechanism of viral replication. Of interest, these structures represent potential targets for the development of new antiviral strategies. Very recently, studies focusing on the dynamics of these organelles using real-time fluorescence microscopy in infected cells revealed that IB assembly could be mediated through a process of liquid-liquid phase separation (LLPS) (9, 26, 42). Such a mechanism was initially described for the formation of cellular membrane-less organelles (45–47). LLPS is initiated by scaffold molecules that form condensates though the establishment of a network of interactions that also allow the partition of client molecules, more frequently proteins and RNA. One described archetype of protein architecture sustaining the formation of LLPS relies on proteins with intrinsically disordered regions (IDRs) presenting multiple interacting motifs of low affinity (43, 48–50). Of note, many IDR-containing proteins present RNA-interacting domains.

Concerning MNV IBs, it is now recognized that expression of the viral N and P proteins is necessary and sufficient to induce the formation of IBs. Indeed, similarly for RABV (26), VSV (42), measles virus (9), RSV (19), MPV (51), or PIV3 (15), the coexpression of these two proteins is sufficient to induce the assembly of IBs. N and P proteins of MNVs present common characteristics such as structural similarities for the globular RNA-binding domain of N and the presence of IDRs in P. However, depending on the virus family, the mode of interaction between N and P, as well as the oligomeric state of P, which is a dimer in the case of rhabdoviruses (52, 53), or a tetramer for paramyxoviruses and pneumoviruses (54, 55), differ. Nonetheless, both N and P are always in an oligomeric state, which is crucial for LLPS.

Here, our aim was to characterize the elements of RSV N and P required for the assembly of IBs. We previously identified the P binding sites on both N^Nuc^ (36, 37) and on monomeric N^0^ (34, 39). We also showed that interaction between N and the C terminus of P is required for IB assembly (37). However, the role of monomeric N as well as the domains of P necessary for the LLPS process remained unknown. Using the N mutant K170A/R185A, which was previously shown to be deficient for RNA binding but still capable of interacting with P (34), thus mimicking the N^0^ form, we first showed that the interaction between RSV P and the monomeric N is not sufficient to induce the assembly of IBs in transfected cells (Fig. 1). A similar approach was previously used to study the formation of MeV IBs (9). In this case, the authors reported that coexpression in cells of MeV P and a N mutant unable to bind RNA, which could mimic N^0^, resulted in the formation of IBs of comparable size and morphology as those observed upon expression of wild-type N. This discrepancy of results could be more related to a difference in the propensity of the N proteins of MNVs to oligomerize. Indeed, it is now admitted that oligomerization of N proteins is closely related or coupled to their ability to interact with RNA (29). Recent biochemical studies on the capacity of recombinant RSV and MeV monomeric N to interact with RNA and oligomerize revealed that, whereas MeV monomeric N can form *in vitro* nucleocapsids in the presence of RNA of 6 nucleotides in length (56), the RSV monomeric N assembles only into rings under the same conditions (57). Altogether, these results suggest that, more than a direct role of RNA in IB assembly, the oligomerization of N seems crucial for the scaffold involved in the formation of IBs. However, it is expected that RNA plays a role in the LLPS process, as described in the literature (43, 48–50).

In parallel, we investigated the minimal elements of P required for the morphogenesis of IBs in RSV. Our first approach was to study the capacity of deletants of P to induce IBs when coexpressed with N in cells. We showed that the OD and C-terminal part of P are essential and sufficient to generate cytoplasmic puncta similar to those observed in the presence of wild-type P, whereas the N-terminal part is not required (Fig. 2). We then assessed the potential role for IB morphogenesis of the conserved region [161-232] of P, which separates the OD and the last 9 C-terminal residues of P and which was recently shown to present a potential secondary P binding site to N (33) and to interact with L (31, 32). We revealed that deletion of this domain impaired IB formation, whereas substitution of this region by a short linker consisting of five repetitions of the Gly-Ser sequence still allows us to observe cytoplasmic IB-like structures (Fig. 4). These results suggest that more than a direct role of residues [160-227] in IB morphogenesis, this domain mainly acts as a flexible region allowing the organization of N-P interactions. However, further investigation will be required to determine whether the P[160-227] in its entirety is optimal for the assembly of IBs and whether this domain is involved in accessory interactions required for the dynamics of IBs.

Finally, we demonstrate the ability of recombinant fluorescent N^Nuc^ and P to drive liquid-liquid phase separation *in vitro*, resulting in the formation of pseudo-IBs (Fig. 5 and 6). FRAP experiments showing that mCherry-N presents similar mobility within *in vitro* IBs compared to pseudo-IBs observed in cells expressing N and P confirmed that *in vitro* IBs can constitute a simplified model for further investigation of the nature and the physico-parameters sustaining the formation and the dynamics of these structures. To our knowledge, this is the first time that IB-like structures can be reconstituted *in vitro* with RSV N and P proteins. It is noteworthy that the P/N molecular ratio for formation of droplets has to be ≥4. Considering the tetrameric state of P, this suggests that one P tetramer could interact with one N protomer, but it also could have multiple contact sites with N. However, using isothermal calorimetry, we have previously shown that one tetramer of P interacts with two N^Nuc^ protomers (34). These data suggest that in addition to N-P interaction, the concentration of P tetramers could be critical for the optimal scaffolding of IBs. Similar droplets were observed in the presence of N and the P[127-241] fragment. This result correlates with those obtained in cells and shows that the N-terminal IDR of P is not directly involved in the scaffold necessary for IB assembly, although we cannot exclude the possibility that this domain of P could modulate the nature and dynamics of IBs during viral infection. Using the mutant of P with the last C-terminal residue (F241) deleted, which is critical for N-P interaction, we also confirmed that this interaction is critical to initiate droplet assembly.

In the course of writing this article, Guseva et al. managed to reconstitute MeV IBs *in vitro* by incubation of recombinant monomeric N with P protein (58). Similar to our findings on RSV P, the authors showed that the P OD and C-terminal domain of MeV are required and sufficient to induce LLPS in the presence of N but also that the C-terminal IDR of P could be involved in the dynamics of IB assembly. As previously mentioned, the role of the corresponding domain of RSV P will need to be addressed.

Altogether, our results, supported by recent data from the literature, clearly indicate that N and P of MNVs are the key elements driving IB assembly and that their interactions are at the origin of the scaffold sustaining the architecture of IBs. However, it appears crucial to combine complementary cellular and *in vitro* approaches in order to fully understand IB morphogenesis and dynamics during viral infection. Analyzing the impact of posttranslational modifications (PTMs) of N and P on IB dynamics would also be of great interest as PTMs are known to modulate other membrane-less organelle assembly as well as the recruitment of client proteins (47). Furthermore, data from the literature already argue for the role of PTMs in nucleocapsid assembly and N-P interaction. Phosphorylation of influenza virus NP would regulate the oligomerization of this protein (59), phosphorylation of the mumps virus N protein was shown to be involved in the regulation of transcription/replication steps (60), phosphorylation of MeV P protein modulates the size of IBs (9), and we recently found that the cellular phosphatase PP1 is recruited by RSV P to IBs and is involved in the phosphorylation/dephosphorylation kinetics of M2-1 (20). Finally, the identification by mass spectrometry of proteins of the methylosome (PRMT5 and WDR77) as partners of N (41) suggests that methylation could regulate the polymerase complex activity, and therefore the dynamics of IBs. Further investigations will thus be required to characterize the potential role of PTMs of both N and P on the composition, nature, and dynamics of IBs.

## MATERIALS AND METHODS

### Plasmid constructions.

Plasmids for eukaryotic expression of the proteins N, N^K170AR185A^ (N^mono^), P, and P-BFP have been described previously (20, 34, 37). The plasmid pmCherry-N was obtained by subcloning the sequence encoding mCherry in fusion with the 5′ end of the N gene. The plasmids pP[1-126] and pP[1-160] were generated by substituting residues 127 and 161 of P by a stop codon, respectively, using the QuikChange site-directed mutagenesis kit (Stratagene). The plasmids pPΔOD, pP[127-241], and pP[161-241] were generated by deletion using the Q5 site-directed mutagenesis kit (New England BioLabs), following the manufacturer’s recommendations. The plasmids pP-BFP-Cter and pP-BFP-gs-Cter were engineered, respectively, by deleting or replacing the 160–227 region by the sequence encoding the stretch GSGSGSGSGS using the Q5 site-directed mutagenesis kit (NEB) (primer sequences available on demand).

For expression and purification of recombinant proteins, the previously described pET-N, pGEX-P, and pGEX-P[127-241] and pGEX-P[161-241] plasmids were used (34). The pET28a-P-BFP was obtained by inserting the sequence encoding the blue fluorescent protein (BFP) sequence between residues 74 and 75 of P, between the NdeI and XhoI sites, using In-Fusion HD cloning kit (Clontech). For expression of P-BFP^ΔF241^, a stop codon was inserted at position 240 of P, using the Q5 site-directed mutagenesis kit (NEB). The pET-mCherry-N expressing the mCherry in fusion with the N terminus of N was obtained by cloning the mCherry sequence at the NcoI site of the pET-N plasmid.

### Antibodies.

The following primary antibodies were used for immunofluorescence and/or immunoblotting: a rabbit anti-P antiserum (40), a rabbit anti-N antiserum (40), a mouse monoclonal anti-N protein (Serotec), a mouse monoclonal anti-HSP70 (Sigma), and a mouse monoclonal anti-β-tubulin (Sigma). Secondary antibodies directed against mouse and rabbit Ig G coupled to Alexa Fluor 594 or Alexa Fluor 488 (Invitrogen) or coupled to horseradish peroxidase (HRP) (P.A.R.I.S) were used for immunofluorescence and immunoblotting experiments, respectively.

### Expression and purification of recombinant proteins.

E. coli BL21 bacteria (DE3) (Novagen) transformed with pGEX-P plasmids were grown in Luria-Bertani (LB) medium containing 100 μg/ml ampicillin. For N purification, bacteria transformed with pET-N or pET-mCherry-N together with pGEX-P[161-241] plasmids were grown in LB medium containing ampicillin (100 μg/ml) and kanamycin (50 μg/ml). For P-BFP expression, bacteria transformed with pET-P-BFP plasmid were grown in LB medium containing 50 μg/ml kanamycin. After incubation of bacteria at 37°C for 8 h, the same volume of LB was then added and protein expression was induced by adding 80 μg/ml isopropyl-β-d-thio-galactoside (IPTG) to the medium. The bacteria were incubated for 15 h at 28°C and then harvested by centrifugation. Purification of glutathione *S*-transferase (GST) fusion protein was performed using the already described protocol (34). To isolate GST-free P proteins, beads containing bound proteins or P-N complexes were incubated with thrombin (Novagen) for 16h at 20°C. Purified recombinant proteins were then loaded onto a Superdex 200 16/30 column (GE Healthcare) and eluted in 20 mM Tris-HCl (pH 8.5) and 150 mM NaCl.

For histidine purification of the recombinant P-BFP, the pellet was resuspended in lysis buffer (20 mM Tris-HCl [pH 8], 500 mM NaCl, 10 mM imidazole, 0.1% Triton X-100,1 mg/ml lysozyme, and complete protease inhibitor cocktail [Roche]). Benzonase (Novagen) was then added to the lysate (final concentration, 5 U/ml), for 1 h at room temperature. After centrifugation, the supernatant was loaded onto a 5-ml HiTrap immobilized metal ion affinity chromatography (IMAC) column (GE Healthcare) charged with 0.2 M NiSO_4_ and equilibrated with low-imidazole buffer (50 mM imidazole, 20 mM Tris-HCl [pH 8.5], 500 mM NaCl) using a 50-ml Superloop. A linear gradient of 50 to 500 mM imidazole in the same buffer was applied to elute His-tagged P-BFP protein. After equilibration with buffer consisting of 20 mM Tris-HCl (pH 8.5) and 150 mM NaCl, P-BFP was further purified by size exclusion chromatography on a HiLoad Superdex-200 column with a 120-ml total bed volume (GE Healthcare).

### Cell culture and transfections.

BHK-21 cells (clone BSRT7/5) constitutively expressing the T7 RNA polymerase (61) were grown in Dulbecco modified essential medium (Lonza) supplemented with 10% fetal calf serum (FCS), 2 mM glutamine, and antibiotics. Cells were transfected using Lipofectamine 2000 (Invitrogen) as described by the manufacturer.

### Fluorescence microscopy.

Cells grown on coverslips were transfected with pN and pP (or plasmids expressing fragments of P). Twenty-four hours after transfection, cells were fixed with 4% paraformaldehyde (PFA) for 25 min. Fixed cells were permeabilized, blocked for 30 min with phosphate-buffered saline (PBS) containing 0.1% Triton X-100 and 0.3% bovine serum albumin (BSA), and then successively incubated for 1 h at room temperature with primary and secondary antibody mixtures diluted in PBS containing 0.3% BSA. For labeling nuclei, Hoechst 33342 (Invitrogen) was added during incubation with secondary antibodies. Coverslips were mounted in Prolong gold antifade reagent (Invitrogen). Cells were observed with a Nikon TE200 microscope equipped with a CoolSNAP ES^2^ (Photometrics) camera or Olympus FV3000 inverted confocal microscope, and images were processed using MetaVue (Molecular Devices) and ImageJ software.

### Expression in eukaryotic cells and soluble/insoluble fractionation analysis.

For protein expression analysis, cells transfected with pN and pP (or mutants) were lysed in Laemmli buffer. For fractionation analysis, cells transfected with pP and/or pN and pN^K170A/R185A^ were washed with phosphate-buffered saline, lysed in lysis buffer (50 mM Tris-HCl [pH 7.4], 2 mM EDTA, 150 mM NaCl, 1% NP-40), and sonicated on ice with a 5-s pulse. After centrifugation at 13,000 rpm at 4°C for 30 min, the supernatant containing soluble proteins and the pellets were recovered and mixed with Laemmli buffer. Samples were boiled, and proteins were resolved by sodium dodecyl sulfate-polyacrylamide gel electrophoresis (SDS-PAGE).

### *In vitro* assay of pseudo-IB formation.

P and N recombinant proteins in buffer consisting of 20 mM Tris-HCl (pH 8.5) and 150 mM NaCl were coincubated at different P/N molecular ratios on glass slides, and the molecular-crowding agent Ficoll was added to the droplets of solution. Then coverslips were laid on the droplets. Pseudo-IBs were then observed with a Nikon TE200 inverted microscope equipped with a Photometrics CoolSNAP ES2 camera. Images were processed using MetaVue software (Molecular Devices) and ImageJ software.

### Time-lapse microscopy and photobleaching experiments.

FRAP experiments were done on *in vitro* IBs obtained by coincubation of mCherry-N and P and on cells seeded on Ibidi μ-Dish polymer coverslip bottom and transfected for 24 h with pmCherry-N and pP. Image acquisition was performed using the Olympus FV3000 inverted confocal microscope in which cells were maintained in a climate-controlled chamber (37°C, 5% CO_2_) during imaging and using the 60× oil immersion objective.

All FRAP experiments were performed using the same settings: 6-s prebleach, 5-ms bleach, and 70-s postbleach at a frame rate of 1 image every 125 ms. Bleaching of mCherry was performed in a circular region at 100% laser intensity. The average fluorescence intensity as a function of the time of every bleached region was obtained using the Icy software. Background intensity was estimated by measuring a region outside the cell as far as possible from the target IB. The quantitative analysis of the recovery curves was performed using the easyFRAP, a MatLab stand‐alone application.
